# One size doesn’t fit all: Should we reconsider the introduction of cold-stored platelets in blood bank inventories?

**DOI:** 10.12688/f1000research.10363.1

**Published:** 2017-02-01

**Authors:** Alessandra Berzuini, Marta Spreafico, Daniele Prati

**Affiliations:** 1 Department of Transfusion Medicine and Hematology, Azienda Socio Sanitaria Territoriale (ASST) di Lecco, Alessandro Manzoni Hospital, Lecco, Italy

**Keywords:** platelet transfusion, platelet concentrates, cold platelets

## Abstract

Platelet concentrates are universally prepared with a standard method and stored for 5 days at room temperature (20–24°C) in gentle agitation. Currently, there is a renewed interest in the possibility of storing platelet concentrates below the standard temperatures. In fact, cold platelets might be more effective in bleeding patients and have a lower risk of bacterial transmission. Inventories including platelets at different temperatures may favour patient-centred strategies for prophylactic or therapeutic transfusions.

Platelet (PLT) transfusion is commonly performed in patients with thrombocytopenia to prevent haemorrhages as well as in patients bleeding from surgical or traumatic injuries. The effects of PLT transfusion, unlike those of red blood cell transfusion, are not fully predictable, and bleeding endpoints in clinical trials are imprecise. As a matter of fact, post-transfusion PLT count is a measure of the circulating compartment, irrespective of the magnitude of PLT eventually involved in mending vascular injuries. Neither PLT function nor interactions with other factors involved in the maintenance of the bleeding/thrombotic equilibrium can be precisely defined, and strong evidence demonstrates that the amount of infused PLTs does not increase post-transfusion efficacy ^1^. For these reasons, there is a general agreement to initiate prophylactic transfusion when the PLT count drops below 10,000, but owing to the lack of studies indicating the required PLT amount, both primary and secondary haemostasis are generally treated with a standard PLT dose of 3×10 ^11^, roughly corresponding to 25% of the PLT mass of a typical adult.

Post-transfusion outcome may be influenced by several factors: firstly, recipient’s characteristics, and, in a certain proportion, blood manipulation may be implicated as well.

Procedures required for the preparation of platelet concentrates (PCs) may impact on the quality of product. PLTs are stressed along tube passages, centrifugations, manipulations, suspension in chemical storage medium, encasement in plastic bags, continuous tumbling and prolonged storage. In compliance with current standard practices, PCs are stored in gas-permeable containers and maintained at 20–24°C (room temperature [RT]) in constant gentle agitation. Time storage is limited to 5 days in order to reduce metabolic decline and to lower the risk of bacterial contamination, which is reported around 1:1,500 PC and is probably an underestimation. Unfortunately, limited storage leads to the wastage of approximately a quarter of inventories. Bacterial quality controls (BQCs) are applied on PCs randomly or universally, depending on different national regulations. Blood culture is the reference method, although new, faster methods have been recently validated. Blood cultures are time-consuming and expensive and somewhat unreliable because they are too long, and in the case of a positive result, a late patient look-back is the only possible intervention. In Europe, universal BQC is not mandatory and is generally applied on random outdated PCs with the aim being to control the system (and not the single bag).

Cryoconservation of PCs (−80°C) is allowed by European and Italian directives ^2– 4^. Frozen inventories are still present in some settings and have very useful applications such as storage of rare phenotypes, autologous or matched PCs for immunised patients, or for rescue inventories. Thawed PLTs have been demonstrated to be very effective in reducing bleeding in surgical patients and are successfully used in military medicine for battlefield injuries. Frozen PLTs have the major advantage of being able to be stored for years. Nevertheless, thawing procedures are time-consuming (approximately 20 minutes), making this product less attractive for hospital emergency situations.

## “Cold platelets”

A less intense refrigeration (4°C) was applied in the USA, with US Food and Drug Administration approval, during the ’60 and ’70s. Ideally, refrigeration has two main advantages: it allows a longer storage and may reduce the risk of bacterial contamination. Nevertheless, cold preparations were soon abandoned because of their shorter survival in transfused patients. This was seen as a limit especially in patients requiring prophylactic transfusions—accounting for 70% of PC recipients—who need a long-acting product. However, in patients with active bleeding due to major trauma or surgery, a promptly more active product might be preferable. Recently, several articles have reconciled the previous studies with new evidence, comparing functional profiles of RT- and cold-stored PLTs. Because control of bleeding in battlefield injuries is a cornerstone of military medicine, most studies come from this scientific community. Accumulating evidence from *in vitro* and *in vivo* studies indicate the opportunity to reconsider the introduction of refrigerated PLTs in blood bank inventories.

## “ *In vitro*” studies

Since the early ’70s, comparative studies on PCs stored at RT and 4°C have identified, for each storage method, some advantages and disadvantages. Although there are no direct measures of PLT function, several indicators have been employed: PLT count, morphology, dosage of glycoproteins and metabolites associated with prolonged storage, expression of activation markers, and functional tests such as aggregometry and clot formation ^5^ ( Table 1).

**Table 1.** The most used measurements of platelet function.

Parameters associated with storage lesions	Parameters associated with activation	Parameters associated with aggregation
pH	Morphology	Rotation thromboelastometry (ROTEM) evaluation
Visible swirling	P-selectin	ADP
Shape change	CD-40L	Collagen
Hypotonic shock recovery	GMP-140	TRAP (thrombin receptor-activating peptide), ristocetin, and arachidonic acid response
Lactate production	GPIB, GPIX, GPIIB, GPIV, and activated IIb-IIIa (PAC-1 binding)	
Glucose consumption	CD62P and CD63	
Cytokines	Annexin V-lactadherin binding	
Microparticles		

Some comparative studies were performed to evaluate how PLT content may be influenced by different storage conditions. Reddoch *et al*. evaluated single-donor PLTs stored for 5 days at different temperature conditions ^6^. Compared with the standard method, cold-stored PCs displayed a median 20% reduction of PLT count. Getz *et al*. made similar observations, but differences disappeared when plasma was replaced with platelet additive solution (PAS) medium ^7^. The reason for this probably resides in the lesser extent of aggregate formation consequent to the dilution of plasma fibrinogen and the lower activation of fibrinogen receptors. Nonetheless, 4°C-PAS-stored-PLT, when compared with RT controls, displayed a better aggregation response to multiple agonists ^7^.

Many authors observed, in 4°C PLTs, the increase of some surface markers such as P-selectin, GMP-140, and CD40L that are associated with an increased level of activation ^8– 12^. During storage, PLTs undergo a gradual loss of PLT quality dependent on various metabolic, functional and morphologic derangements, globally defined as storage lesions, which are expected to increase when PLTs are stored at RT ^13^. PLT degranulation is one of the typical changes induced by RT storage and is characterised by lysis of dense bodies and alpha granuli, lysosome secretions, shedding of proteins and membrane vesiculations known as microparticles. Several studies tried to identify laboratory measures associated with the metabolic decline: some of them are not difficult to achieve during the daily management of blood inventories, such as pH, PLT count and the swirling phenomenon (that is, the shimmering appearance of viable PLTs when exposed to a light source) ^14^. Reddoch *et al*. reported, at 5 days of storage, acceptable pH levels for Cold-stored Aliquota; nevertheless, in cold PCs, the swirling phenomenon had disappeared. This change is not necessarily a negative feature, because it may be attributed to the spherical shape typical of activated PLTs. The authors also measured the amount of pro-inflammatory mediators possibly related to apoptotic degranulation (such as sCD40L and TxA2) and found them significantly increased in RT PCs ^6^. These results are in agreement with Baimukanova *et al*., who, in RT-stored PCs, found an accumulation of those inflammatory factors—cytokines, chemokines and growth factors—that are known to play a role in inducing vascular leaks. Since PLTs normally exert a stabilising effect on endothelium, an increase of these metabolites is expected to reduce the vascular integrity and to increase endothelial permeability. To explore this field, Baimukanova *et al*. also tested the effect of differently stored PLTs on transendothelial resistance and vascular permeability of a mouse cell monolayer. As expected, 4°C-stored PLTs exhibited a greater capacity to inhibit endothelial permeability than RT-stored PLTs ^15^. PCs stored at different temperatures have been evaluated for a long time to individuate different aggregation behaviours. The first evaluation was by Becker *et al*. ^16^, who with *in vitro* functional experiments in the early ’70s observed that 4°C-stored PCs had increased aggregation properties. Reddoch *et al*., testing PLT adhesion properties on injured endothelium, observed, in RT PLTs only, a reduced adhesion to collagen-coated channels, thus suggesting a temperature-mediated PLT dysfunction or a degradation of plasma coagulation factors ^6, 17^. Over and above, Baimukanova *et al*., testing PLT with aggregation agonists—ADP, collagen and thrombin receptor-activating peptide (TRAP)—found that cold PLTs produced a faster, stronger and longer-lasting clot ^15^. We find corresponding evidence in a study by Getz *et al*. ^7^, who, by means of flow cytometry, detected in refrigerated PCs an increment of spontaneous PLT aggregation. Johnson *et al*. ^18^ observed in cold PCs a faster thrombin generation and measured an increased amount of microparticles (fragments of membrane vesciculation, supposed to have a haemostatic effect). Alving *et al*. ^19^, testing aggregation properties of 4°C PCs, registered that they had retained almost 90% of their functionality until 21 days of storage when tested in aggregation studies *.* Reddoch *et al*. sustain the hypothesis that cold-stored PLTs are primed in a pre-activation state but at the same time maintain the innate responsiveness to stimuli ^6^. This is the picture of slightly activated PLTs, more efficient in aggregation and adhesion functions and competent to form a stronger clot ^9^.

This evidence raises a logical concern over a theoretical risk of a prothrombotic action of cold-stored PLTs. Conversely, Reddoch *et al*. have demonstrated that 4°C-stored PLTs display a normal response to physiologic inhibitors and that aggregation was hindered by natural antagonists, such as nitrous oxide and prostaglandin I _2_ (PGI _2_), which are commonly released by injured endothelium ^17^. Of importance, in the literature, there are no reports of thrombotic episodes in patients transfused with refrigerated PLTs.

Of practical importance, Reddoch *et al*. observed that for cold PLTs there was no influence of constant agitation on either parameters or functions, thus indicating that the requirements of refrigerated PLTs are easier and cheaper ^6^. Table 2 reports a summary of several study results describing an increased expression of activation markers and aggregation properties of 4°C PCs compared with the standard RT storage.

**Table 2.** List of *in vitro* experiments comparing platelet storage at 4°C versus room temperature.

Authors	Journal	Year	4°C versus room temperature
Johnson *et al*. ^18^	*Transfusion*	2016	Reduction of glycolysis Increased expression of P-selectin Faster thrombin generation Faster clot formation, equal strength
Bynum *et al*. ^22^	*Transfusion*	2016	Less oxidative stress Stronger clot Increased response to aggregating agents Better aggregation in shear stress conditions
Getz *et al*. ^6^	*Transfusion*	2016	No difference in platelet content in the first 5 days of storage No difference in rotation thromboelastometry (ROTEM) pattern after 5 days of storage
Wood *et al*. ^10^	*Transfusion*	2016	Decreased expression of GPIB, GPIX, GPIIB, and GPIV (easier von Willebrand factor attack) Increased expression of P-selectin, tetraspanin, and phosphatidylserine Cytoskeleton protein modifications corresponding to activation state (proteomics) Increased expression of CD62P, CD63, and annexin V
Baimukanova *et al*. ^15^	*Transfusion*	2016	Increased aggregation potential
Reddoch *et al*. ^9, 17^	*Shock* *Shock*	2014 2016	Increased expression of CD40 and P-selectin Increase of intracellular free calcium Increase of dense granule release of ATP Accelerated thrombin generation More pronounced response to ADP, collagen, and TRAP (thrombin receptor-activating peptide) Faster, stronger, and more durable clot
Mondoro and Vostal ^23^	*Platelet*	2002	Increased response to ADP and epinephrine Stronger clot resistance to disaggregating agents No spontaneous aggregation
Connor *et al*. ^12^	*Transfusion*	1996	Reduced expression of GMP-140 ADP response of 250%; collagen response of 100% at more than room temperature
Triulzi *et al*. ^11^	*Transfusion*	1992	Increased expression of GMP-140
Rinder *et al*. ^7^	*Transfusion*	1990	Increased expression of GMP-140
Becker *et al*. ^16^	*Transfusion*	1983	More pronounced ADP response

The main results about platelet marker expression and platelet functionality are reported.

## “ *In vivo*” studies

*In vivo* experiments began in the early ’70s with comparative studies by Valeri and Becker *et al*. over the ability of PCs stored at different temperatures to stop bleeding. Valeri reported a reduced post-transfusion lifespan of cold PLTs ^20^, and the same results were obtained by Becker *et al*., who found that cold PLTs after 24 hours of storage survived for 2–4 days (which was significantly inferior to the 8–10 days of RT PLTs). Nevertheless, Becker *et al*. found no significant difference in PLT increment when fresh ^51^Cr-tagged PLTs (within 24 hours of storage at both temperature conditions) had been transfused in a hundred patients, and the authors also found a moderate but significant superiority in PLT increment in patients receiving fresh cold PLTs. This trend took the opposite direction after 48 hours of storage. Concurrently, the two sets of authors found evidence of a preferential clearance of activated PLTs after transfusion, hypothesising the recognition of P-selectin by the mononuclear-phagocyte system ^16^. Nevertheless, Valeri also observed that refrigerated PCs, unlike RT-stored PCs, did reduce the bleeding time in aspirin-treated healthy volunteers, and Becker *et al*. observed a better efficiency of cold PLTs in reducing the bleeding time of patients with thrombocytopenia ^8, 16, 20^. Very recently, Strandenes *et al*. planned a two-arm randomised pilot study to evaluate the efficacy of PLTs cold-stored up to 7 days in the treatment of post-operative bleeding in patients undergoing cardiothoracic surgery. Preliminary results of a comparative analysis with PLTs stored under standard conditions indicate equal efficacy of cold-stored PLTs with a trend towards less post-operative bleeding. Nonetheless, PLT aggregation was higher in patients who received cold PLTs ^21^.

## Conclusions

A great deal of evidence suggests that, besides having a shorter circulation time, PCs stored at 4°C might have better haemostatic competence and a better safety profile in terms of infectious risks. An important issue is the possibility of extending shelf life without lowering functionality. Large and well-designed clinical trials are required to decide whether cold PCs should be introduced in clinical practice for some indications in PLT transfusion.

Refrigerated PCs also have organisational advantages, as cold storage overcomes the need for constant agitation and reduces storage space and number and the need for BQCs, improving cost profile.

Cold PLTs may be integrated in the blood bank organisation as a manageable product suitable to provide timely availability in rural settings, mountainous territories, islands, and battlefields; to support difficult rescue operations; and to provide valid support to trauma centres. The availability of cold PCs, in addition to those maintained at RT, may limit the precautionary overproduction of blood components, thus reducing wastage.

## Abbreviations

BQC, bacterial quality controls; PAS, platelet additives solution; PC, platelet concentrate; PLT, platelet; RT, room temperature; TRAP, thrombin receptor-activating peptide.
